# Co-occurrence of behavioral risk factors of common non-communicable diseases among urban slum dwellers in Nairobi, Kenya

**DOI:** 10.3402/gha.v8.28697

**Published:** 2015-09-16

**Authors:** Tilahun Nigatu Haregu, Samuel Oti, Thaddaeus Egondi, Catherine Kyobutungi

**Affiliations:** 1 African Population and Health Research Center, Nairobi, Kenya; 2 Department of Global Health, Academic Medical Center, University of Amsterdam, Amsterdam, The Netherlands; 3 Amsterdam Institute for Global Health and Development, Amsterdam, The Netherlands

**Keywords:** non-communicable diseases, risk factors, co-occurrence

## Abstract

**Background:**

The four common non-communicable diseases (NCDs) account for 80% of NCD-related deaths worldwide. The four NCDs share four common risk factors. As most of the existing evidence on the common NCD risk factors is based on analysis of a single factor at a time, there is a need to investigate the co-occurrence of the common NCD risk factors, particularly in an urban slum setting in sub-Saharan Africa.

**Objective:**

To determine the prevalence of co-occurrence of the four common NCDs risk factors among urban slum dwellers in Nairobi, Kenya.

**Design:**

This analysis was based on the data collected as part of a cross-sectional survey to assess linkages among socio-economic status, perceived personal risk, and risk factors for cardiovascular and NCDs in a population of slum dwellers in Nairobi, Kenya, in 2008–2009. A total of 5,190 study subjects were included in the analysis. After selecting relevant variables for common NCD risk factors, we computed the prevalence of all possible combinations of the four common NCD risk factors. The analysis was disaggregated by relevant background variables.

**Results:**

The weighted prevalences of unhealthy diet, insufficient physical activity, harmful use of alcohol, and tobacco use were found to be 57.2, 14.4, 10.1, and 12.4%, respectively. Nearly 72% of the study participants had at least one of the four NCD risk factors. About 52% of the study population had any one of the four NCD risk factors. About one-fifth (19.8%) had co-occurrence of NCD risk factors. Close to one in six individuals (17.6%) had two NCD risk factors, while only 2.2% had three or four NCD risk factors.

**Conclusions:**

One out of five of people in the urban slum settings of Nairobi had co-occurrence of NCD risk factors. Both comprehensive and differentiated approaches are needed for effective NCD prevention and control in these settings.

According to the World Health Organization (WHO), non-communicable diseases (NCDs) accounted for about 36 million deaths globally in 2008. That was about 63% of 57 million total deaths in the same year (1). The Global Burden of Diseases, Injuries, and Risk Factors Study 2010 (GBD 2010) estimated that deaths due to NCDs increased from 57% of total mortality in 1990 to 65% in 2010 (2, 3). About 80% of global deaths related to NCDs occur in low- and middle-income countries (LMICs). These countries also have a high proportion of premature deaths due to NCDs. LMICs account for 90% of the 9 million premature NCD-related deaths (before age 70 years) (4, 5).

The four common NCDs (CVD, cancers, diabetes, and chronic respiratory diseases) which account for 80% of the NCD burden share four common risk factors. These are unhealthy diet, insufficient physical activity, harmful use of alcohol, and tobacco smoking (6). Along with the rapid changes in lifestyles and increasing impacts of globalization and urbanization, the magnitude of NCD risk factors is also increasing in many LMICs. This has been followed by the rapid emergence of many NCDs in those settings (7).

Accordingly, progress has been made in generating research-based evidence and formulating policies that address the epidemic of NCD globally and in many LMICs (8). Research on the prevalence of NCDs and their risk factors, such as WHOSTEP surveys, is a key example of some of the progress being made in generating evidence relevant to NCDs (9). However, such evidence is very limited in sub-Saharan Africa, especially among the urban poor settings. Besides, most of the existing evidence is based on ‘single-factor’ and ‘single-disease’ approaches and has neglected the co-occurrence of NCDs and their risk factors (10). Despite the knowledge that the NCD risk factors are shared, one risk factor could lead to multiple NCDs and one NCD could be a result of multiple risk factors, there has been little attempt to investigate the co-occurrence of the common NCD risk factors in Africa. ‘Single-factor’ or ‘single-disease’ approaches may lead to vertical programs which may be very useful in specific contexts but may be unsustainable in the long term (11, 12). In this regard, diabetes projects in many LMICs are key examples of disease-specific approaches. Critics of such approaches have called for more integrated, comprehensive, and coordinated approaches that could strengthen health systems and respond to multiple conditions or risk factors (13).

Evidence on the co-occurrence of risk factors could inform coordinated approaches to addressing them from a health system perspective. For instance, in individuals with harmful use of alcohol and smoking, an integrated counselling on both of these risk factors would be efficient and effective. Similarly, obesity is a result of combined effects of unhealthy diet and insufficient physical activity. Integrated interventions that can address obesity would involve both diet and physical activity interventions. Evidence on the level of co-occurrence of unhealthy diet and insufficient physical activity would be critical for such interventions.

In Kenya, where the urban population constitutes 24% of the total population, the probability of dying between age of 30 and 70 years from the four major NCDs is 18%. In this country, NCDs were estimated to account for about 27% of total deaths. Recent statistics also show that Kenya has a current tobacco-smoking rate of 13%, total alcohol per capita consumption of 4.3 l, and obesity prevalence of 4.3% (14). In the slum areas, precarious housing sub-markets whose physical forms vary by place and time but uniformly characterized by inadequate provision of basic infrastructure and public services necessary to sustain health, such as water, sanitation, and drainage (15), there is little information about the patterns of co-occurrence of NCD risk factors. Using the existing data, this study aims to determine the prevalence of co-occurrence of the four common NCD risk factors (unhealthy diet, insufficient physical activity, harmful use of alcohol, and tobacco use) among urban slum dwellers in Nairobi, Kenya. The resulting evidence is expected to inform the development and implementation integrated of comprehensive, multi-sectoral interventions that acknowledge the co-occurrence of risk factors among individuals in the context of urban poor settings in sub-Saharan Africa.

## Methods

### Data source

The Nairobi Urban Health and Demographic Surveillance System (NUHDSS) was set up to provide a platform to investigate the inter-linkages between urban poverty and health; monitor and evaluate intervention programs implemented within the study communities and provide a sampling frame for nested studies. NUHDSS runs in two slums of Viwandani and Korogocho in Nairobi, Kenya. This demographic surveillance area is home to over 72,000 people resident in about 30,000 households. Details of NUHDSS have been published elsewhere (16, 17).

The assessment of the linkages among socio-economic status, perceived personal risk, and risk factors for cardiovascular and related NCDs (also called the ‘cardiovascular disease [CVD] study’) was a population-based, cross-sectional survey conducted by the African Population and Health Research Center (APHRC) within the NUHDSS population between May 2008 and April 2009. This study utilized the sampling frame from the NUHDSS: a stratified, sampling strategy based on the WHO STEPwise protocol with a target of 250 respondents in each of the following strata: sex, age group (18–24, 25–30, 31–40, 41–50, 51–60, and 60+), and slum of residence (Korogocho and Viwandani). Data were collected from a total of 5,190 individuals aged 18 years and above. Additional details of the sampling strategy, data collection methods, and data quality assurance mechanisms of the CVD study are published elsewhere (18).

### Definitions

Data on socio-demographic, behavioral, and physiological risk factors for CVD and other relevant NCDs were collected based on WHO STEPS approach for chronic disease risk factor surveillance. The four common NCD risk factors used in this study are defined in Table 1.

**Table 1:** Definitions of the four NCD risk factors used in this analysis

S. no.	NCD risk factor	Definition used in this study
1	Unhealthy diet	Consumption of less than five portions of fruit and vegetables a day on at least 5 days on an average week and/or greater than six teaspoon of sugar a day (19)
2	Insufficient physical activity	Less than 75 min of vigorous-intensity physical activity or less than 150 min of moderate-intensity physical activity per week or equivalent of combinations of these (from work, walking/cycling and leisure) (20)
3	Harmful use of alcohol (based on daily consumption)	More than three standard units/day for men; more than two standard units/day for women – above low risk for developing alcohol use disorder (21, 22)
4	Tobacco use	Current tobacco use as self-reported by the respondent (23)

*Co-occurrence* of NCD risk factors was defined by the existence of two or more (out of the four) NCD risk factors in an individual at the time of the survey. It included dyads (two risk factors), triads (three risk factors), or the presence of all the four risk factors in an individual respondent.

#### Measurements

Basic socio-demographic variables and NCD risk factors were assessed using a structured, pre-tested and interview administered questionnaire. Data collected on NCD risk factors included information about diet, physical activity, smoking, and alcohol. For diet, the questionnaire, detailed questions about consumption of fruit and vegetables, sources of fat, and sugar intake were included. The physical activity section assessed work-related, walk/cycling-related, and recreational/sports-related physical activities. Alcohol units were converted into standardized units using show cards for the different types of alcohols. In the smoking section, data on ever smoking, current smoking, daily smoking, duration of smoking, and type of tobacco products used were collected.

### Statistical analysis

We conducted a descriptive analysis of the basic socio-demographic characteristics of the study population using proportions. The average daily consumptions of fruit and vegetables in a week, estimated from the number of days fruit and vegetables are consumed and the number of servings in a typical day, were used to calculate the prevalence of unhealthy consumption of fruit and vegetables. This was then combined with sugar consumption to estimate the prevalence of unhealthy diet. Physical activity time was calculated from minutes spent in a week for work related, walk or cycling, and recreational/leisure-related physical activities. The average number of standard units of alcohol consumed in a day was used to estimate the prevalence of harmful use of alcohol. A summative scoring of the risk factors was used to estimate the prevalence of co-occurrence of different combinations of the risk factors.

Sampling probability weight was computed using the size of the stratum in the NUHDSS database as denominator and response probability was calculated using the total number sampled per stratum as denominator. A composite weight taking both sampling and response weights into account was applied to all prevalence estimates.

Key analyses were stratified by sex, age, and other relevant socio-demographic variables. Chi-square statistics was used to assess binary associations between categorical variables. Associations between the NCD risk factors were assessed using logistic regression analysis. Data were analyzed using SATA 12. *P*-values<0.05 were considered to be statistically significant.

### Ethical considerations

The study protocol was approved by the Kenya Medical Research Institute/National Ethical Review Committee (NON-SSC Protocol No. 339). Participants provided written consent to participate in the study. The participants who accepted to be interviewed had signed the consent form to show that they accepted to participate in the study. The Ethics committee has approved the consent procedure along with the protocol and data collection tools.

## Results

### Background characteristics

A total of 5,190 study participants were included in this analysis. Of these 2,794 (53.8%) were men and the rest 2,396 (46.2%) were women. Nearly 56% of the study participants were married and 25% were widowed at the time of the survey. The highest level of education for nearly a quarter of the study population was less than primary school and for 42% of them it was primary school. About 42% of the study participants were involved in small businesses to generate their income, while 15% were not working at the time of the survey. The age–sex distribution of the study population has been reported elsewhere (24).

### Prevalence of NCD risk factors

#### Unhealthy diet

Nearly half (48.6%) of the respondents eat fruit for not more than 3 days a week. Only one-third (33.2%) of the study population reported that they eat fruit 7 days a week. About 65% of them reported that they eat vegetables throughout the week. The prevalences of low consumption of fruit and vegetables, defined by less than five average daily servings on most days of the week (5/7) were 94 and 61%, respectively. The prevalence of lower consumption of both fruit and vegetables (added together) was 57% (63% in women and 53% in men) and the prevalence of low consumption of either of them was 39.4%. On the other hand, the prevalence of high daily consumption of sugar (defined as more than six teaspoons of sugar a day) was 18.9% (22.4% in women and 15.8% in men). The weighted prevalence of unhealthy diet (as defined by inadequate consumption of fruit or vegetables and/or high sugar intake) was 57.2% (95% CI: 55.8%, 58.5%).

#### Insufficient physical activity

For about 20 and 40% of the respondents, their work involved vigorous-intensity physical activity and moderate-intensity physical activity, respectively. The work of 566 (11%) respondents involved both vigorous- and moderate-intensity physical activity. Almost all participants did walking or cycling, and about 55% did it throughout the week. Less than 5% of the study population reported involvement in sport-related physical activity. Analysis of the contribution of the different forms of physical activity to the total physical activity time in the total study population shows that about 70% of the physical activity came from work-related activities and 27.6% from walking or cycling. Only 1.6% came from sports.

The weighted prevalence of insufficient physical activity, defined by less than 75 min of vigorous-intensity physical activity or less than 150 min of moderate-intensity physical activity per week or equivalent of combinations of these, in the study population was 14.4% (95% CI: 13.5%, 15.4%). This was significantly higher in women (25.9%) than in men (6.9%).

#### Harmful use of alcohol

Of the 5,190 study participants, 849 (16.4%) reported that they have ever consumed alcohol. Of these, 32% take at least one drink for 1–3 days a month, while 29% had alcohol 1–4 days a week. Eighteen percent had less than once a month. Only 111 (13%) reported daily consumption of alcohol. The mean (SD) number of units of alcohol consumed in a single day among those who ever consumed alcohol was 3.5 (2.0). The weighted prevalence of harmful use of alcohol, defined by more than three standard units of alcohol per day for men and more than two standard units of per day for women, was 49.5% among those who ever consumed alcohol and 10.05% (95% CI: 9.25%, 10.9%) among the total study population.

#### Tobacco use

About one-fifth (20.3%) of the study participants have ever smoked. The weighted prevalence of current smoking among the study population was 12.4% (95% CI: 11.5%, 13.3%). The weighted prevalence of current daily smoking among current smokers was 18.9% in men, 0.7% in women, and 11.7% in the total study population.

**Fig. 1:**
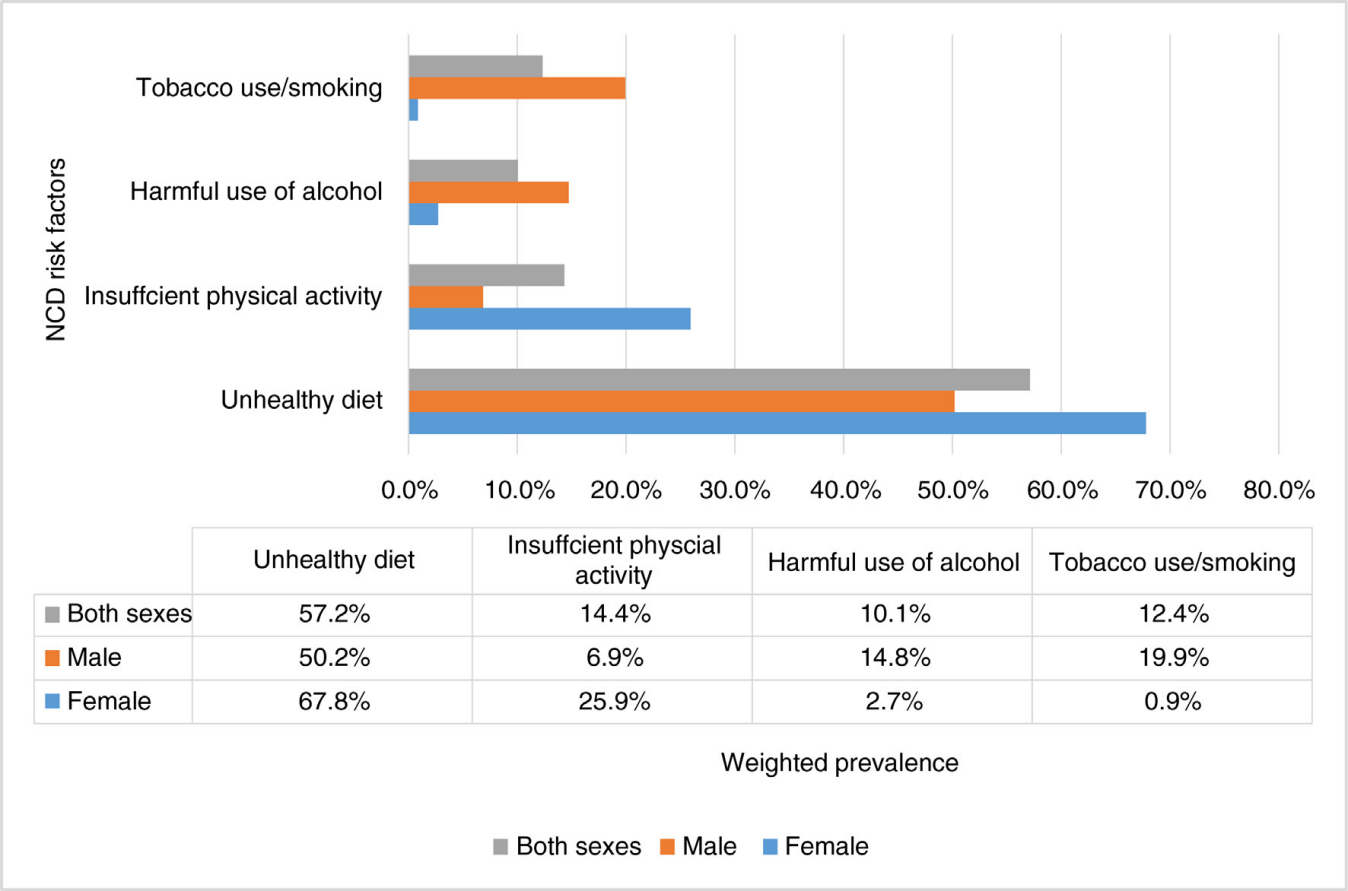
Weighted prevalence of the four common NCD risk factors by sex.

Disaggregated analysis of prevalence the NCD risk factors by sex showed that unhealthy diet and insufficient physical activity were higher among women, while harmful use of alcohol and tobacco use were higher among men. A summary of the weighted prevalences of the four common NCD risk factors is presented in Fig. 1.

### Co-occurrence of NCD risk factors

#### At least one of the four NCD risk factors

Of all the study participants, 3,739 (72.06%: 95% CI: 70.8%, 73.3%) had at least one (i.e. one or more) of the four common NCD risk factors. Only 1,450 (27.94%) were free from all the four common NCD risk factors. The prevalence of at least one NCD risk factor was higher among women than men (76.8% vs. 67.9%; *X*^2^=51.4, *P*=0.000).

#### Any one of the four NCD risk factors

A little more than half, 2,708 (52.2%), of the study participants had a single NCD risk factor (i.e. any one of the four common NCD risk factors). Among those who had a single NCD risk factor, the prevalence of unhealthy diet, insufficient physical activity, harmful use of alcohol, and tobacco use were found to be 78.5, 11.5, 3.7, and 6.2%, respectively. The prevalence of any one of the four NCD risk factors among women (57.97%) was significantly higher than that of men (47.23%). The pattern of single risk factors was consistent across age groups.

#### Dyads (two risk factors)

In this analysis, 914 (17.6%) of participants had dyads – two NCD risk factors. The combination between unhealthy diet and insufficient physical activity was the most common dyad. This dyad had a significantly higher prevalence in women (17.3%) than in men (4.7%). The prevalence of dyads was higher among those aged 60+ (20.7%). It was lower among those between 40 and 50 years of age (14.4%). Those study participants who were not working at the time of the study had the highest prevalence of dyads (25.4%) as compared to those who were working (16.2%).

#### Three or more risk factors

The co-occurrence of three or more NCD risk factors was found in only 113 (2.2%) study subjects. Among the triads, the combination of unhealthy diet – alcohol – tobacco use had higher prevalence. Only four (0.08%) of the study population had all four common NCD risk factors (see Table 2).

**Table 2:** Prevalence of NCD risk factors by number of factors

S. No	Number of risk factors	Number	Percent (95% CI)
1	No risk factors (0/4)	1,450	27.9% (26.7%, 29.2%)
2	One risk factor (1/4)	2,708	52.2% (50.8%, 53.5%)
3	Two risk factors (2/4)	914	17.6% (16.6%, 18.7%)
4	Three risk factors (3/4)	113	2.2% (1.8%, 2.6%)
5	Four risk factors (4/4)	4	0.08% (0.02%, 0.2%)
6	Two or more risk factors	1,031	19.9% (18.8%, 20.9%)

### Predictors of co-occurrence of NCD risk factors

Men were found to be at a higher risk of having co-occurrence (the presence of two or more) of the common NCD risk factors as compared to women (OR=1.28; 95% CI: 1.11, 1.49). Those who were not working (assess by yes/no question for currently working or not) at the time of the survey also had a higher probability of having co-occurrence of NCD risk factors as compared to those who were working (OR=1.81; 95% CI: 1.49, 2.21). Similarly, those above the age of 50 years were about 1.2 times more likely to have co-occurrence of NCD risk factors as compared to those less than or equal to 50 years of age. However, educational status (ever attended school vs. never attended school) (OR=1.21; 95% CI: 0.97, 1.51) did not have any significant association with co-occurrence of NCD risk factors.

### Association between NCD risk factors

Examination of the associations among the four common NCD risk factors indicated that there was a strong positive association between harmful use of alcohol and tobacco use. Unhealthy diet had a negative association with harmful use of alcohol and tobacco use. There was also a moderately positive association between tobacco use and insufficient physical activity. All other binary associations between the NCD risk factors were not statistically significant. Details of these associations are presented in Table 3.

**Table 3:** Association between the four common NCD risk factors (adjusted for age, sex, educational status, and working status)

S. no.	Combination of two risk factors (dyads) (First factor outcome, second predictor)	Adjusted odds ratio	95% CI
1	Unhealthy diet and insufficient physical activity	0.93	(0.79, 1.09)
2	Unhealthy diet and harmful use of alcohol	0.65	(0.52, 0.80)
3	Unhealthy diet and tobacco use	0.82	(0.68, 0.98)
4	Harmful use of alcohol and tobacco use	5.52	(4.36, 7.00)
5	Harmful use of alcohol and insufficient physical activity	1.07	(0.76, 1.51)
6	Tobacco use and insufficient physical activity	1.18	(0.87, 1.59)

## Discussion

The accelerating burden of NCDs in developing countries is now a major public health concern and a medical challenge. In this regard, the co-occurrence of NCDs is associated with sub-optimal health outcomes and at the same time with rising healthcare expenses for patients and health systems (25). Among a number of factors contributing to the co-occurrence of NCDs is the co-occurrence of NCD risk factors in adults.

In this study, we found that nearly 75% of the study population had at least one of the four common NCD risk factors. Unhealthy diet was remarkably high and may account for the majority of the NCD risk in these urban slum settings. About one out of five individuals had co-occurrence of common NCD risk factors at the time of the survey. This is highly significant in an African context where infectious diseases and maternal health problems are the main priorities. In a study conducted in Bangladesh, clustering of NCD risk factors was 38% (26). It may also reach 70% in rural Asian populations (27). Prevalence of clustering in Florianopolis was 43% in men and 37% in women (28).

Co-occurrence of common NCD risk factors – tobacco use, harmful use of alcohol, insufficient physical activity, and unhealthy diet – contribute substantially to NCD prevalence. For instance, about 17% of the study population from a sample of 29,183 subjects in the United States had three or more risk factors (29). High prevalence of multiple and clustered behavioral risk factors underline the challenge comorbidity presents for primary care and public health systems (29). Similarly, the prevalence of multiple behavioral risk factors was considerably high in this sample of European adults. Studies have shown that those who were not living alone and had higher education may prove protective (30). In comparison, in our study we have found a prevalence of 20% of co-occurrence NCD risk factors. Females were at an increased risk of having multiple NCD risk factors as compared to men. Those who were older also have greater prevalence of multiple risk factors. A study conducted in Brazil had also reported that higher age and low socio-economic status was associated with clustering of NCD risk factors (31).

As it is shown in the Results section, the association between harmful use of alcohol and smoking was very strong. Those study participants who smoke were about 5.5 times more likely to be engaged in harmful use of alcohol as compared to those who were not smoking. This was consistent with findings from other studies in different parts of the world (32–34). This could be due to the interplay among various socio-behavioral factors that affect both smoking and drinking behavior.

In a study that assessed individual, social, and school correlates of multiple chronic disease behavioral risk factors (insufficient physical activity, sedentary behavior, tobacco smoking, alcohol drinking, and high body mass index) in a representative sample of Canadian youth aged 10–15 years attending public schools, several individual and social characteristics were associated with the co-occurrence of multiple behavioral risk factors; however, no school-related correlates emerged. Knowledge about correlates of multiple behavioral risk factors should be considered when planning prevention programs (35). In this regard, age, sex, and working status were main correlates of co-occurrence of multiple NCD risk factors in our study population.

Behavioral risk factors are known to co-occur in many population groups. Their co-occurrence has the potential to increase risks of chronic disease comorbidity and increased mortality in later life. In general, little is known about determinants of multiple chronic disease behavioral risk factors, particularly in low- and middle-income settings. Despite the fact that most of the previous studies were cross-sectional in their nature and/or were carried out without a sound theoretical framework, evidence suggests that targeting individual/social distal variables in prevention programs of multiple chronic disease behavioral risk factors could be effective (36). It was also recommended that prevention programs should take into account differential distribution of lifestyle risk factors (37). Similarly, future interventions against NCD risk factors in urban slum settings need to differentially target those with multiple NCD risk factors. Evidence from that study suggested that combined package interventions should target elder population, men, and rural residents, especially those with lower socio-economic status (38). Thus, the findings of this study could contribute to that body of knowledge about NCD risk factors for urban poor settings in Africa.

WHO attributes approximately 3 million deaths a year from NCDs to inadequate consumption of fruit and vegetables (39). A study estimated that increased consumption of fruit and vegetables is associated with a 16% lower risk of CVD. To address this problem, increasing the availability of good-quality seeds, encouraging the farmers to grow vegetables and fruit, and a public-awareness campaign about their benefits are some of the important strategies that may help increase the consumption of fruit and vegetables (40). The findings from this study substantiate these recommendations.

The co-occurrence of NCD risk factors also has important implications in the performance of individual-level risk assessment tools (WHO/ISH charts). It has demonstrated that data from STEPS survey which were used to perform risk assessment at individual level could present results of the CVD risk at population levels.

There were some limitations associated with this study. In the estimation of the prevalence of unhealthy diet, we used the consumption of fruit, vegetables, and sugar. We have not used consumption of fats and salts as data on these were lacking. In the measurement of time for physical activity, respondents reported their time in hours and this may be less precise. For tobacco use, we used current smoking as a proxy indicator. We have not taken into consideration frequency, duration, and dose of smoking. Moreover, our definition of co-occurrence differs from clustering and a direct comparison of findings with other studies needs to consider this difference. Finally, the measurement of all four risk factors was self-reported and thus may not be free from social desirability bias. Interpretation of the findings needs to take these limitations into account.

## Conclusions and recommendations

Among the four common NCD risk factors, unhealthy diet had the highest prevalence in the study area. More than two-thirds of the study population had at least one NCD risk factor and about one-fifth had co-occurrence of NCD risk factors. Females and the elderly were at a greater risk of having co-occurrence of NCD risk factors. While the dyads of unhealthy diet and insufficient physical activity were more common in women, the dyads of harmful use of alcohol and tobacco use were more common in men.

As the prevalence of co-occurrence NCD risk factors was significantly high in the study population, and some population groups were at increased odds of having co-occurrence of NCD risk factors, future interventions need to adopt the approach of ‘combined but differentiated’ interventions. Further research is needed to elucidate the additional correlates and possible impacts of co-occurrence of multiple NCD risk factors. Research and surveillance of NCD risk factors need to factor in the co-occurrence of NCD risk factors in their design and analysis of results.
